# A Comprehensive Review of In-Body Biomedical Antennas: Design, Challenges and Applications

**DOI:** 10.3390/mi14071472

**Published:** 2023-07-21

**Authors:** Khaled Aliqab, Iram Nadeem, Sadeque Reza Khan

**Affiliations:** 1 Department of Electrical Engineering, College of Engineering, Jouf University, Sakaka 72388, Saudi Arabia; 2 Department of Information Engineering and Mathematics Science, University of Siena, 53100 Siena, Italy; iram.nadeem@student.unisi.it; 3 Institute of Sensors, Signals and Systems, School of Engineering and Physical Sciences, Heriot-Watt University, Edinburgh EH14 4AS, UK

**Keywords:** biocompatibility, capsule endoscopy, frequency band, implantable, ingestible, injectable, SAR

## Abstract

In-body biomedical devices (IBBDs) are receiving significant attention in the discovery of solutions to complex medical conditions. Biomedical devices, which can be ingested, injected or implanted in the human body, have made it viable to screen the physiological signs of a patient wirelessly, without regular hospital appointments and routine check-ups, where the antenna is a mandatory element for transferring bio-data from the IBBDs to the external world. However, the design of an in-body antenna is challenging due to the dispersion of the dielectric constant of the tissues and unpredictability of the organ structures of the human body, which can absorb most of the antenna radiation. Therefore, various factors must be considered for an in-body antenna, such as miniaturization, link budget, patient safety, biocompatibility, low power consumption and the ability to work effectively within acceptable medical frequency bands. This paper presents a comprehensive overview of the major facets associated with the design and challenges of in-body antennas. The review comprises surveying the design specifications and implementation methodology, simulation software and testing of in-body biomedical antennas. This work aims to summarize the recent in-body antenna innovations for biomedical applications and indicates the key research challenges.

## 1. Introduction

**Figure 1:**
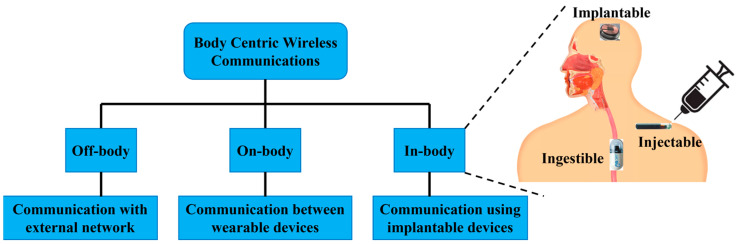
Types of BWCS.

Body-centric communication system (BWCS) is an emerging technology referring to human self and human-to-human networking, which uses implantable and wearable sensors [1]. BWCS is a combined field of wireless body area networks (WBANs), wireless personal area networks (WPANs) and wireless sensor networks (WSNs). It is also classified as off-body, on-body and in-body communication, as shown in Figure 1. On-body communication is the communication between different wearable devices. The communication between an outside and an on-body device is designated as off-body communication [2]. In-body communication system means the communication of implantable devices and sensors inside the body with an external device or communication with another implant. In-body biomedical devices (IBBDs) are designed to monitor physiological data inside the human body and provide key support to improve the quality of life through disease prevention, therapy and diagnosis, such as drug delivery system, neurostimulators, bone growth stimulators, and treatment of numerous severe conditions in the medical profession [3]. Wireless IBBDs are divided into implantables, ingestibles and injectables based on the way they are inserted into the human body [4]. Specifically, implantable devices are the most common type of IBBDs sited inside the human body through a surgical operation [5]. In the last decade, implants have advanced from bulky pacemakers to micro-sized deep brain implants [6]. Ingestible devices are generally capsule-shaped devices, which are ingested and swallowed, similar to regular pills [7]. The most conventional ingestible device is the wireless endoscopic capsule, which was originally discovered in the year 2000 [8]. Currently, wireless ingestible capsules are equipped with cutting-edge abilities, which can also monitor the side effects of pharmaceuticals [9].

Lastly, injectable devices [10] are micro-sized devices, which can be injected into the human body using needles. Recently, these devices have been commonly used for important sensing and neurostimulation applications [10]. The primary components of an IBBD are the antenna, battery, the processing system and sensors [11]. The reliability and strength of the wireless link between the external and internal device largely depend on the antenna mounted on an IBBD. Therefore, the antenna is a key construction block of an IBBD, as the primary working requirement of signal reception and transmission depends mainly on the performance of the antenna. Furthermore, the overall size and weight of the IBBD can also be affected by it.

In IBBDs, the antenna is primarily used for wireless communication and data transfer [12,13], wireless power transfer (WPT) [14] and sensing [15,16], which lead to a wide range of medical applications, including continuous pressure measurements, dental antenna for remote healthcare, intracranial pressure monitoring, glucose level check, insulin pump, radiometer/heating therapy, pacemaker connection, endoscopic capsule and blood pressure measurements [17].

The design of an in-body antenna is challenging, as it is mostly situated in electromagnetically harsh and lossy environments inside the human body. The electromagnetic (EM) wave passing through the lossy heterogeneous tissue inside the human body can cause significant absorption of most of the antenna radiations [18]. Such inhomogeneous human organ structure is the primary reason for the impedance mismatch, which makes the radiation performance inefficient. This also affects the antenna efficiency significantly [19]. The powering of an in-body antenna attached to an IBBD inside the human body is another key research challenge. The commonly used batteries in IBBDs are bulky in size with limited capacity and can complicate the system design process [20]. Recently, in-body antennas for WPT have become a great research interest [14]. WPT necessitates an appropriate selection of the frequency band, which is a vital component of the in-body antenna system for biomedical applications. The operation frequency band of an in-body antenna must avoid EM interference with the current terrestrial frequency bands [21]. Furthermore, the fabrication and testing of such miniaturized in-body antennas inside the human body are extremely challenging due to the inadequately accredited animal laboratories, along with major health and safety related issues [22,23].

The growing research in this area demands a comprehensive overview in order to acquaint the new researchers and antenna designers with the state of the art and current developments. This review work aims to describe the design specifications, implementation and testing techniques, challenges and different applications of in-body antennas. Following the Introduction, the paper is divided into six sections. Section 2 briefly highlights the design specifications of in-body antennas. Section 3 explains the in-body antenna design, manufacturing and testing process. Section 4 outlines the different challenges in the development of in-body antennas. Section 5 details the different antenna types being used in different IBBD applications and compares their performances critically. Section 6 briefly discusses the limitations of the current in-body antenna designs and indicates future research scopes. Finally, the conclusions are drawn in Section 7.

## 2. Design Specifications

### 2.1. Operation Frequency Bands

**Figure 2:**
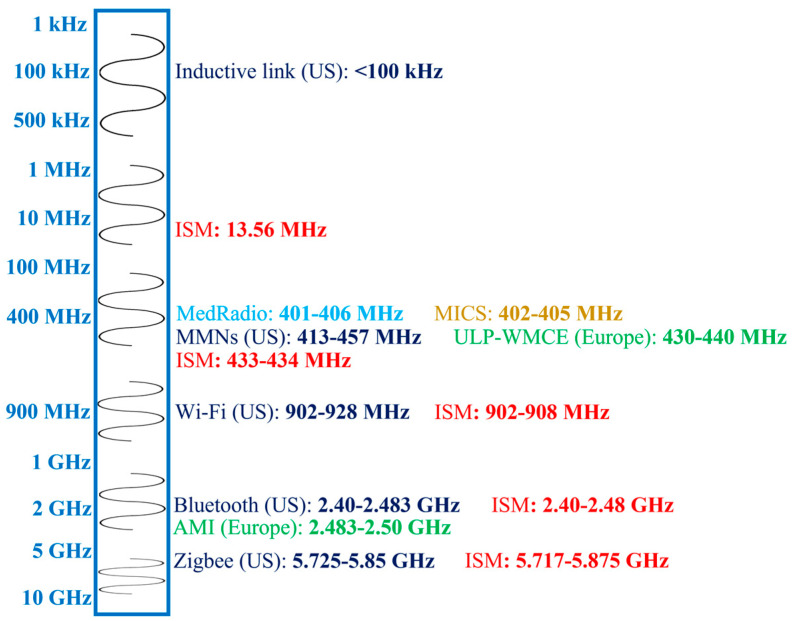
Frequency bands for in-body antennas.

Figure 2 shows different frequency bands allocated for in-body antennas. The choice of an operation frequency for IBBDs involves several trade-offs. Generally, lower frequencies are attractive and commonly used, as they facilitate lower loss of the biological tissue medium, which can lead to higher efficiency and better tissue safety. However, lower frequencies have the drawback of limiting the communication speed and requiring larger antenna size. In contrast, higher frequency bands provide high data rates and miniaturization at the cost of lower tissue safety. In Figure 2, frequencies below 100 kHz are allocated for short-range inductive IBBDs by the Federal Communication Commission (FCC) in the United States (US) for lower power and data transmission [21]. Medical micropower networks (MMNs) are another FCC-approved short-range frequency band with a 24 MHz spectrum from 413 to 457 MHz. Furthermore, Wi-Fi (902–928 MHz), Bluetooth (2.40–2.483 GHz) and Zigbee (5.725–5.850 GHz) are designated for short-range digital modulation communication for IBBDs by the FCC in the US.

In Europe, the Electronic Communication Committee (ECC) allocated the 430 to 440 MHz band for ultra-low-power wireless medical capsule endoscopy (ULP-WMCE) and the 2.483 to 2.50 GHz band for active medical implants (AMI). The Medical Device Radio Communication Service (MedRadio) band of 401–406 MHz [4], Medical Implant Communication System (MICS) band of 402–405 MHz [24] and Industrial, Scientific and Medical (ISM) band of 13.56 MHz, 433–434 MHz, 902–908 MHz, 2.40–2.48 GHz and 5.717–5.875 GHz [25] are accepted worldwide for IBBD application.

### 2.2. Miniaturization

Antenna miniaturization is a key specification for IBBDs. In MedRadio, MICS and common ISM bands, the effective size of the in-body antenna at the desired resonance frequency becomes significantly larger. This can create difficulties during the implantation process of IBBDs in human tissue [2]. Therefore, the size of in-body antennas is considered very crucial, and miniaturization techniques, such as changing the physical properties of the structure, varying the material characteristics or introducing supplementary elements, are used to solve these kinds of challenges.

#### 2.2.1. High Permittivity Dielectric Substrate or Superstrate

High permittivity substrate/superstrate material can shift the antenna resonant frequency near to the lower frequency band, which shortens the operation wavelength [2]. This simple technique can provide a higher degree of miniaturization. Some of the materials, which are generally used as a dielectric substrate for in-body antennas, include alumina ceramic (relative permittivity, ε_r_ = 9.4) [26,27] and Rogers 3210 [28], 3010 [29], 6002 [30] with ε_r_ = 10.2. A substantial reduction in effective antenna length is realized by using MgTa_1.5_Nb_0.5_O_6_ as a dielectric substrate, with the dielectric constant ε_r_ = 28 [31]. However, this method results in a significant level of surface wave excitation within the substrate. This results in lower bandwidth and a decrease in overall radiation efficiency [32]. The higher cost of these materials is another issue.

#### 2.2.2. Path Lengthening of Current Flow

By modifying the physical properties of an in-body antenna, it is possible to attain a prolonged path of effective current flow. This shifts the resonant frequency to a lower band, resulting in significant size reduction of the antenna [2,17]. Numerous design techniques are considered for this purpose, such as meandered [33], spiral [33], hook slotted [3], waffle type [30] and radiator staking methods [27]. This technique can suffer from higher ohmic loss, resulting in lower radiation efficiency [32].

#### 2.2.3. Impedance Matching with Loading

In-body antennas commonly require the matching of impedance at the anticipated frequency of operation using loading techniques. The loading can possibly be inductive or capacitive, which can effectively minimize the imaginary part of the impedance by nullifying the effect of reactance, helping in size reduction. In Ref. [29], a circularly polarized implantable patch antenna was presented, where the size was reduced due to the use of capacitive loading compared to traditional square patch antenna. However, the impedance matching of a high-quality-factor (*Q*-factor) in-body antenna can lead to performance issues and may require a separate compensation network. This can also reduce the bandwidth of the antenna.

#### 2.2.4. Pin Shorting

Introducing a shorting pin between the patch planes and the ground increases the effective size of the antenna, resulting in a reduction of the essential physical dimensions of it for an explicit frequency of operation [2,3]. This is a similar technique, where the ground plane doubles the height of a monopole antenna. Therefore, it generally produces a planar inverted-F antenna (PIFA) with identical resonance performance, similar to a double-sized antenna deprived of the shorting pin [34]. However, it can cause a reduction in antenna aperture, resulting in a significant decrease in antenna directivity, which can affect the effective gain of the antenna directly [32].

#### 2.2.5. High Frequency Band

The use of a higher frequency band can reduce the size of the in-body antenna significantly. Higher frequencies of operation have shorter wavelengths, which leads to a decrease in antenna size [2,17]. Alternatively, if the antenna size is reduced, the resonant frequency of the antenna will move to a higher band and vice versa. Higher frequencies with a wide bandwidth are also desirable for better data communication [35]. However, they suffer from higher tissue attenuation and loss compared to lower frequencies, which affect the overall in-body antenna performance by inducing more losses [36]. It is also necessary to maintain the operation frequency band specification, as described in Section 2.1.

#### 2.2.6. Modification of Ground Plane

In-body antennas can also be miniaturized by modifying their ground plane. Generally, the model of an in-body antenna considers infinite ground plane. In practice, the ground plane is designed as finite. For large-scale miniaturization, the size of the ground plane is further reduced in a way where, at times, it is slightly larger than the dimensions of the patch [32]. Refs. [37,38,39] present an analysis of truncated ground planes. Such miniaturization can be achieved by introducing a slot in the ground plane, which can alter the return path of the current to slow down the current flow. This causes a phase shift of the displacement of the current from one edge of the slot to the other, resulting in a smaller antenna size [40]. However, such in-body antenna has a reduced polarization concentration, and reducing the size of the ground plane can also affect input impedance. Furthermore, the edge diffraction due to ground plane modification can generate significant back lobe radiation, resulting in a reduction in front-to-back ratio [32].

#### 2.2.7. Use of Metamaterial

Metamaterials are defined as artificially engineered materials, which are designed to provide material properties that are not commercially available to satisfy any extraordinary conditions [41]. They can also be engineered to achieve materials with close-to-zero values of permittivity, negative permittivity or permeability, or simultaneous negative permittivity and permeability. Therefore, they can dramatically reduce the in-body antenna size and can also improve its bandwidth and gain [42]. In Ref. [43], a circularly polarized in-body antenna could achieve 84% size reduction by using a metamaterial design. Although the use of metamaterials has been effective in reducing antenna size, there is a substantial cost in terms of using a complex material, significantly narrow operating bandwidths and lower radiation efficiencies [32]. Additionally, in metamaterial-based miniaturization methods, substantial care must be taken with the analytical models, which are used for the analysis. These analytical models typically ignore the polarization of the field, which might cause different behaviors as compared with the regular incidence or non-polarized models commonly used for analysis to calculate the effective medium properties.

### 2.3. Wireless Link Consideration

**Figure 3:**
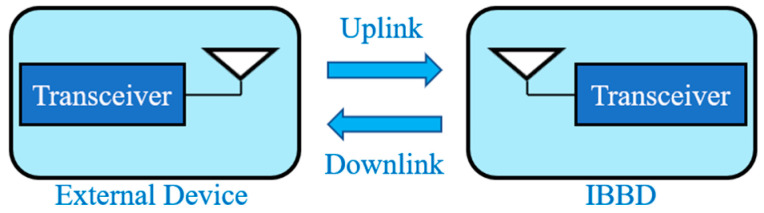
Wireless communication link between external device and IBBD.

Figure 3 shows a generalized wireless communication link between an external device and an IBBD with a transceiver system and an antenna on each side.

The wireless communication link can be classified as near field and far field. The near field technique includes inductive coupling [14]. It was the technology integrated into the first wireless implants at an operation frequency of 20 MHz or lower [4]. This technology established the use of inductors within IBBDs and external devices, which were located in close proximity to initiate wireless communication through coupling. The link design strategies for inductively coupled IBBDs are explained broadly in Refs. [14,44]. In far field, the link power budget can be written as [29]

(1) $$Link\;margin\;\left(dB\right)=Link\frac{C}{N_{0}}-Required\;\frac{C}{N_{0}}\;\begin{array}{l} =P_{t}+G_{t}-L_{f}-L_{imp}+G_{r}-N_{0}-\frac{E_{b}}{N_{0}}-10{log}_{10}B_{r}\\ +G_{c}-G_{d} \end{array}$$

where $C/N_{0}$ is the ratio of the carrier power and noise density; *P_t_*, *G_t_*, *L_f_*, *G_r_*, *B_r_*, *G_c_*, *G_d_* and $E_{b}/N_{0}$ are the transmit power, transmit antenna gain, path loss (free space), receiver antenna gain, receiver bit rate, receiver coding gain, receiver deterioration and the ratio of energy per bit and noise density, respectively. The path loss can be calculated according to the free-space reduction in signal strength with the distance *d* between the transmitter and receiver as [29]

(2) $$L_{f}\;\left(dB\right)=20log\left(\frac{4\pi d}{\lambda }\right)$$

Furthermore, the impedance mismatch loss can be calculated as

(3) $$L_{imp}\;\left(dB\right)=-10log\left(1-{\left|\Gamma \right|}^{2}\right)$$

where Γ is the appropriate reflection coefficient. The loss in human body is not considered in Equation (1), and this will be explained further in Section 4, where different challenges for in-body antenna design are described.

### 2.4. Powering

IBBDs have been conventionally powered via batteries [11,45]. However, integration of the batteries can upsurge the size of the IBBDs, raising biocompatibility and patient safety related concerns. This also necessitates the requirement for frequent battery replacement and/or recharging due to short lifetime. Therefore, the research on battery-less techniques, such as energy harvesting and WPT, for IBBDs is becoming necessary. Energy harvesting technologies involve harvesting the power from environmental or human bodily sources. The motion of the tissue and heartbeat [46], body thermal gradients [47], human movement and motion [48], and glucose oxidization [49] are some of the mechanisms used in the past to harvest the energy for IBBDs. Different WPT techniques and their design methodologies for IBBDs are explained in Ref. [14]. Widespread research has been carried out in the last decade to improve the efficiencies of the aforementioned approaches and develop them to be used for powering IBBDs.

### 2.5. Biocompatibility

In-body antennas are installed in human bodies, so they must have biocompatible properties in order to satisfy patient safety. If in-body antennas are directly embedded into the human body, the body is short circuited due to the fact that human tissues are conductors. Therefore, as a measure to prevent such undesirable short circuit cases, biocompatibility becomes necessary for extended-term implantation of in-body antennas. Two types of methods are mostly proposed for biocompatibility issues of implantable antennas [3]. The first approach is to use a biocompatible substrate for antenna manufacturing, and the second approach is to cover the implantable antenna with a thin coating layer of biocompatible low-loss material. The biocompatible superstrate materials proposed for in-body antennas are Teflon, MACOR and Ceramic Alumina [34]. However, it is problematic to drill and assemble round cuts in ceramic substrates [50,51]. The materials proposed for antenna coating are PEEK [52], Zirconia [53], biomedical-grade-based Silastic MDX-4210 elastomer [51]. A significantly slim layer of low-loss biocompatible material coating increases the properties of biocompatibility in in-body antennas. However, a cautious design of the in-body antenna is required to avoid any performance dependencies related to the thickness of the biocompatible layer [54]. An improvement in the biocompatibility of in-body antenna coating Perylene C material on both sides of the antenna is also proposed in Ref. [55]. The electromagnetic properties of Zirconia make it a better contender for bio-encapsulation [3]. Its significantly lower loss tangent and higher permittivity value help decrease the power loss by accumulating the near field of the antenna inside the capsulation. Additionally, the benefit of PEEK and Silastic MDX-4210 is that they offer simple fabrication processes and are easy to handle.

### 2.6. Safety Consideration

The maximum allowable power incident in the in-body antenna is limited by issues related to patient safety. The specific absorption rate (SAR), which represents the amount of energy deposited per unit mass of tissue, is usually accepted as the most suitable scientific measure in compliance with international guidelines. The IEEE C95.1-1999 standard confines the average SAR over any 1 g of tissue in the shape of a cube to less than 1.6 W/kg (SAR_1g, max_ ≤ 1.6 W/kg) [56], which is followed by the FCC in the US. The international commission on non-ionizing radiation protection (ICNIRP) standardizes the limit of SAR averaged over 10 g of contiguous tissue to be less than 2 W/kg [57]. To comply with ICNIRP guidelines, the IEEE C95.1-2005 standard limits the average SAR over any 10 g of tissue in the shape of a cube to less than 2 W/kg (SAR_10g, max_ ≤ 2 W/kg), which is followed by the European Union [58].

## 3. Antenna Design, Manufacture and Testing

**Figure 4:**
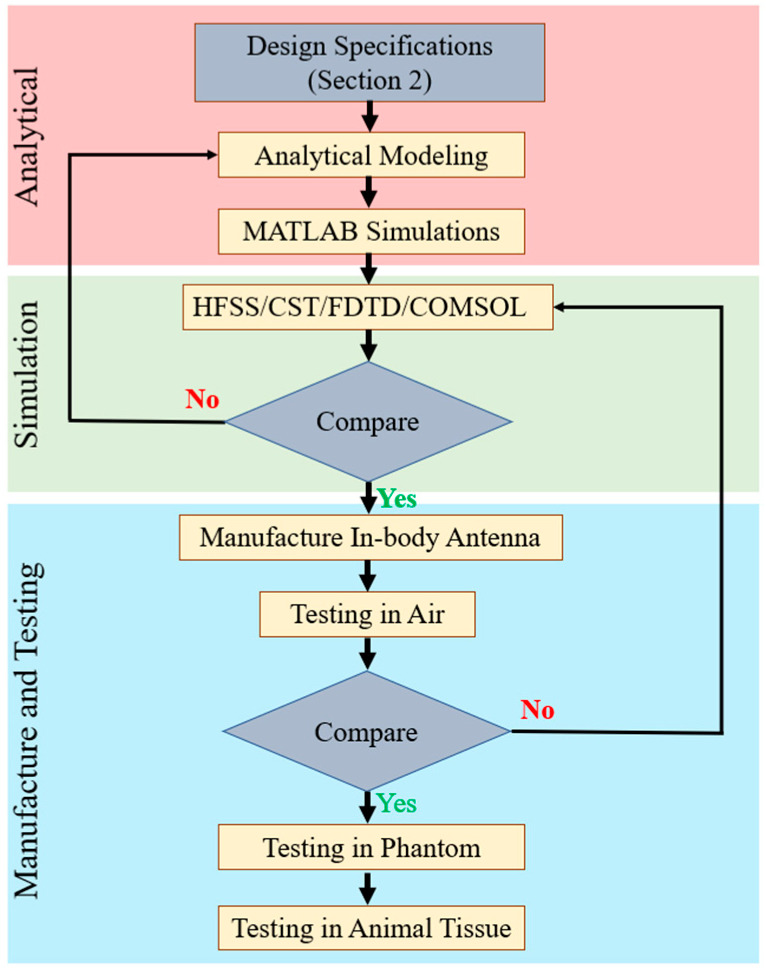
Generalized steps for designing, manufacturing and testing in-body antennas.

Figure 4 shows the generalized steps for designing, manufacturing and testing in-body antennas. In the first step, the researchers benchmark the antenna parameters based on the specifications provided in Section 2. These parameters are then used to generate an analytical model, which leads to analysis and simulation in programming and numeric computing platforms, such as MATLAB [44,59,60,61,62,63,64]. This is an important step of optimizing the antenna to accomplish the best performance [65].

In the next step, the data generated from the analytical model are used to build a 3D antenna simulation environment using electromagnetic (EM) software, such as Ansys high frequency structure simulator (HFSS) [66] https://www.ansys.com/en-gb/products/electronics/ansys-hfss (accessed 15 May 2023), Dassault Systems CST microwave suite [67] https://www.3ds.com/products-services/simulia/products/cst-studio-suite/ (accessed 15 May 2023), finite difference time domain (FDTD) [68] https://optics.ansys.com/hc/en-us/articles/360034914633-Finite-Difference-Time-Domain-FDTD-solver-introduction (accessed 15 May 2023), Altair FEKO [69] https://altair.com/feko (accessed 16 May 2023) and COMSOL [64] https://www.comsol.com/rf-module (accessed 16 May 2023). Furthermore, Ansys Maxwell 3D [70] and Dassault Systems Simulia are low frequency solvers used to design in-body antennas with lower MHz to kHz frequency ranges. The analytical model and EM software simulation results are compared to verify and confirm the antenna parameters before manufacturing in-body antennas.

Detailed methodology of the manufacturing process of an in-body patch-type planar antenna has been outlined in Ref. [65]. In antenna manufacturing, a photolithography mask is first produced to confirm the antenna geometry, including antenna layers, which are going to be stacked on the plane. In the next step, the antenna layers are etched according to the antenna geometry using the photolithography mask. Furthermore, the lower substrate comprises the ground and lower patch; the upper substrate comprises the upper patch; and the superstrate is positioned on the top. Additionally, a circular-shaped hole is etched as per the patch geometry, where four pins are located at the base of the mask. Afterward, all the layers are machined to the circular format, and the layers of the antenna are positioned in a straight line.

This process must be conducted without putting much more mechanical stress on the antenna. Finally, the layers of the antenna are organized in a mountain format and glued to attach all the layers in case of a multi-layered structure. This step is not required for single-layer microstrip patch planar antenna. In the next step, the shorting pin is attached to the ground plane and lower patch. Therefore, the outer conductor of the co-axial feeding point is connected to the ground. Furthermore, the inner conductor is soldered to the lower and upper patch. This methodology is commonly used in research laboratories to validate the antenna parameters with respect to simulation. However, low-temperature ceramic Co-fire (LTCC) is a popular method used in industrial manufacturing. Helical antennas built with conductive wires are generally wound by hand and coil-winder-machined with a counter for laboratory testing and industrial manufacturing, respectively. The manufactured antenna is first characterized in the air, and the measured results are compared with the EM software simulation outputs. In case of a major discrepancy, the researchers go back to the previous step and reiterate it, as shown in Figure 4. Otherwise, the in-body antenna is tested inside phantom (in vitro) and animal tissue (in vivo) successively.

### 3.1. Testing of In Vitro Antenna

**Figure 5:**
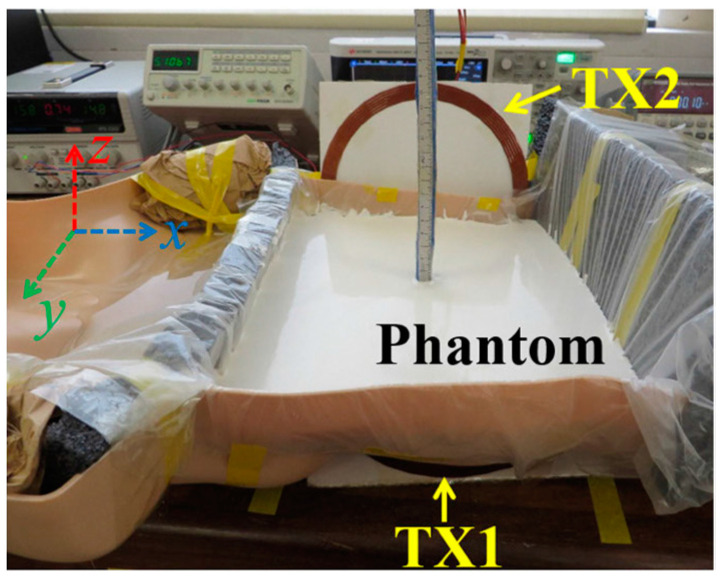
Liquid phantom for capsule localization investigation [70]. Reproduced with permission from an open-access article from *IEEE Access* (CC-BY).

**Figure 6:**
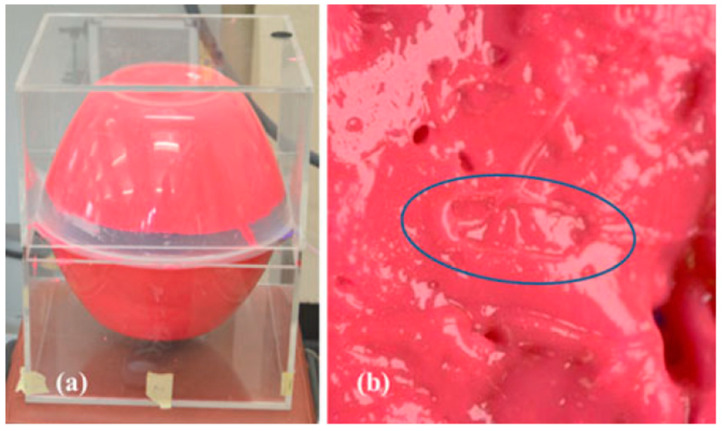
(**a**) Phantom setup. (**b**) Indentation of capsule in phantom [13]. Reproduced with permission from an open-access article from *IEEE* (CC-BY).

The manufactured in-body antenna is verified in the in vitro antenna testing procedure using an artificially built biological environment or phantom [65]. The biological tissue properties (relative permittivity and electrical conductance) of different parts of the body at different operating frequencies are provided in the Foundation for Research on Information Technologies in Society (IT’IS) website, which was established through the resourcefulness and support of the Swiss Federal Institute of Technology (ETH) in Zurich [71]. Before preparing the phantom of a particular body part, it is necessary to know its permittivity and electrical conductance at the in-body antenna operation frequency. Low-frequency band liquid phantom, as shown in Figure 5, is investigated in Refs. [70,72], where purified water, polyethylene powder and NaCl are used as the main material, relative permittivity and conductivity generation material, respectively. Several works presented the investigation of the phantom in the ISM and MICS bands [13,28,73,74]. In Ref. [13], a gel type phantom imitating the properties of muscle tissue is built with hydrophilic organic powder and degassed water, as shown in Figure 6.

A traditional method of measuring the tissue properties of a phantom is the co-axial probe, where a dielectric probe kit, such as SPEAG DAK 3.5, along with a vector network analyzer, such as Agilent 8753ES, are used [75]. An alternative method utilized in the literature involves a dielectric resonator in close contact with the tissue [76]. These measurement techniques utilize the input reflection coefficient to calculate the material dielectric properties. Measurement uncertainty is significant for higher permittivity values, as there is less change in the measured reflection coefficient for discrepancies in material permittivity [75].

### 3.2. Testing of In Vivo Antenna

**Figure 7:**
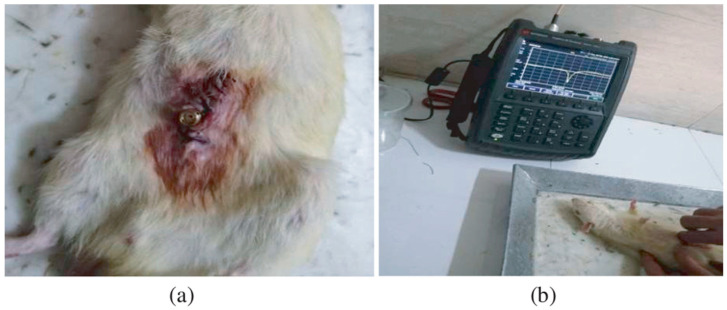
Multi-layer implantable antenna measurement for continuous glucose monitoring. (**a**) A sensor implanted in a rat, (**b**) Experimental setup [77]. Reproduced by courtesy of the Electromagnetics Academy.

**Figure 8:**
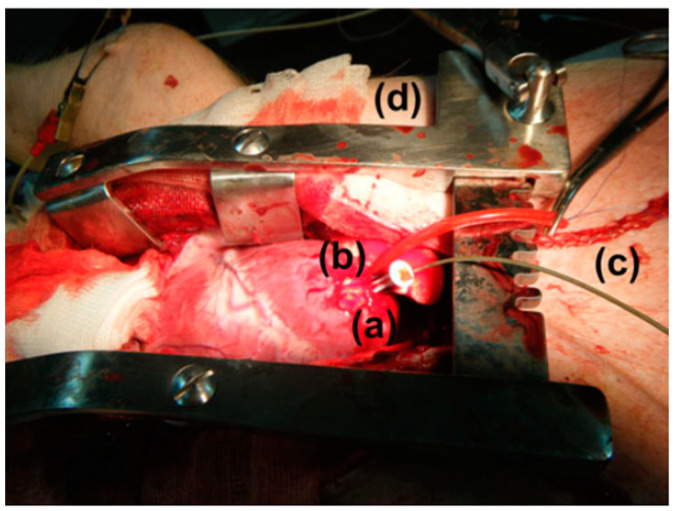
Anesthetized mixed landrace pig with (**a**) exposed left ventricular (LV) apex, (**b**) implanted wireless pressure sensor, (**c**) catheter-tip transducer and (**d**) chest spreader [23]. Reproduced with permission from an open-access article from *Biomedical Microdevices* (CC-BY).

In vitro study is commonly carried out in an artificial biological environment, which cannot confirm the stability of the implanted antenna system because of the lack of dynamic illustration of a real biological environment in the in vitro study [51]. Therefore, the testing of in-body antenna in a real biological environment (in vivo) is commonly suggested after in vitro testing. Before implantation of the in-body antenna prototype inside the biological body, it is necessary to ensure that the temperature of the testing environment is below 100 °C. Generally, the in-body antenna itself generates heat up to 60 °C because of the battery and other internal system devices. Furthermore, the in-body antenna must be insulated by using biocompatible material to protect the antenna system from coupling loss, as described in Section 2.5. Figure 7 and Figure 8 [23,77] illustrate the in vivo testing of the glucose monitoring implantable antenna in a rat and monitoring of blood pressure of the left ventricle, respectively.

## 4. Challenges That Influence the Design of In-Body Antennas

The development of in-body antennas faces numerous design challenges. Miniaturizing the effective electrical size of an antenna leads to a reduction in its electromagnetic performance [78]. Furthermore, it is necessary to consider some important factors to ensure patient safety during the design phase of in-body antennas. First, the in-body antenna is required to be biocompatible, and the SAR must be controlled within the standard limit. This section describes the factors that influence the design specifications of in-body antennas.

### 4.1. Effect of Tissue Diversification

The propagation of a radio wave through the biological tissue is more complex compared to wave propagation in a free space due to the lossy property of the biological tissue causing absorption. Absorption in a radio wave is primarily characterized by considering the permeability, permittivity and conductivity parameters of the medium. An EM wave propagating in the positive *Z*-direction is defined as [79]

(4) $$E\;\left(z\right)={Ee}^{-\gamma z}$$

where *E* and *γ* are the complex amplitude of the wave in the *z*-direction and the complex propagation constant, respectively. *γ* is defined as [79,80]

(5) $$\gamma =j\omega \sqrt{\mu \varepsilon }$$

The constant increase in *γ* leads to attenuation of the electromagnetic wave inside the inhomogeneous region, where *µ* = *µ_r_µ*_0_ = *µ*_0_ defines the permeability of the medium, as for biological materials *µ_r_* = 1. However, the relative permittivity *ε_r_* (where *ε = ε*_0_*ε_r_*) of human body tissue is a complex frequency-dependent parameter, as the conductivity *σ* is not zero. The dielectric properties of the human tissue can be obtained from its relative complex permittivity as [81]

(6) $$\varepsilon_{r}^{*}\left(\omega \right)=\varepsilon_{r}^{\prime }\left(\omega \right)-\varepsilon_{r}^{\prime \prime }(\omega )$$

where $\varepsilon_{r}^{\prime }$ and $\varepsilon_{r}^{\prime \prime }$ are the real and imaginary parts of relative complex permittivity. The imaginary part of relative complex permittivity can be determined from the angular frequency *ω* and conductivity *σ* by

(7) $$\varepsilon_{r}^{\prime \prime }(\omega )=\frac{\sigma }{\omega \varepsilon_{0}}$$

Furthermore, the loss tangent, tan *δ*, which is a measure of how lossy the human body can be, is calculated as follows:

(8) $$\tan\delta =\frac{\varepsilon_{r}^{\prime \prime }}{\varepsilon_{r}^{\prime }}$$

Therefore, as per Equation (7), greater conductivity demonstrates a higher relative complex permittivity value. Furthermore, the increase in the frequency leads to a lower value of imaginary relative complex permittivity in a lossy medium. As a result, the increase in the loss tangent makes the medium significantly lossy. In brief, the complex propagation constant depends on three parameters: permittivity, permeability and conductivity. The increase in conductivity can cause lossy medium, where the radio waves can be significantly attenuated. Therefore, the EM wave attenuates with the increase in the complex propagation constant, as specified in Equation (4).

Another major issue with tissue diversification is that the radio wave propagation speed decreases because of the complex inhomogeneous characteristics of biological tissue. Therefore, the radio wave propagation speed primarily depends on the permittivity and conductivity of the medium. Additionally, the propagation speed in any medium is characterized based on phase (*V_p_*) and group (*V_g_*) velocity as [79,82]

(9) $$V_{p}=\frac{\omega }{\beta }$$

(10) $$V_{g}=\frac{\partial \omega }{\partial \beta }$$

where *β* is the phase constant, defined from the complex propagation constant as [83]

(11) $$\beta =\omega \sqrt{\frac{\mu_{0}\varepsilon_{0}\varepsilon_{r}^{’}}{2}\left(\sqrt{{\left(\frac{\sigma }{\omega \varepsilon_{0}\varepsilon_{r}^{\prime }}\right)}^{2}}+1\right)}$$

In Equations (9) and (10), the propagation speed is characterized based on the phase constant, and it decreases with the increase in the conductivity of the medium. In contrast, the rise in the frequency influences the increase in propagation speed. In summary, higher conductivity of biological tissues can cause significant reduction in the propagation speed of radio waves.

### 4.2. Impact of Effective Wavelength on In-Body Antenna

In Ref. [79], the effective wavelength *λ* in any medium is defined as

(12) $$\lambda =\frac{2\pi }{\beta }$$

The phase constant *β* is dependent on conductivity *σ* proportionally. The rise in medium conductivity reduces *λ,* which therefore leads to miniaturization of the in-body antenna. In an ideal case, in order to facilitate a surgical procedure, the IBBDs have to be in the range of 1 to 10 mm in diameter for a length of 5 to 35 mm, while in the MedRadio and ISM bands, the free-space wavelength is approximately 74 and 12 cm, respectively [78]. This indicates that in-body antennas must be profoundly miniaturized, leading to antenna dimensions of some fractions of the free-space wavelength (typically *λ*/30 and *λ*/5 for the MedRadio and ISM bands, respectively).

### 4.3. Effect of Efficiency

In a free space, the radiated power of an antenna depends on the far field elements only, as the near field is mostly reactive and therefore not distressing the radiated or the absorbed power [78]. In the case of an antenna radiating into lossy matter, the near field component plays a key role by causing strong coupling with the surrounding medium near to the antenna and hence increases the losses. Therefore, the total radiated power primarily depends on the radial distance *r*. The outer boundaries of the near field and far field are commonly assumed as $r<0.62\sqrt{D^{3}/\lambda }$ and $r<D^{2}/\lambda$, where *D* is the antenna’s highest dimension [84]. In the case of in-body antennas, the situation improves slightly because the complex lossy medium surrounding the antenna is of finite dimensions. In the lossy medium, strong coupling with nearby lossy biological tissues is caused by the radiated radio wave. Therefore, the coupling of frequency causes a loss of radiated power. This coupling is also the primary driver of lower radiation efficiency of in-body antennas. Furthermore, biocompatibility encapsulation using insulating materials plays a key role in reducing the coupling with the adjacent lossy environment [78].

### 4.4. Biocompatible Encapsulation

The process of covering the in-body antenna with biocompatible material is known as encapsulation. In Ref. [78], the effect of encapsulation on radiation efficiency is described through a comparative study between Zirconia and PEEK used as encapsulation shells for in-body antennas. It was observed that Zirconia demonstrates better results than PEEK due to its significantly lower loss and higher dielectric constant, which agrees with a higher concentration of the near field in the low-loss surrounding of the in-body antenna. Furthermore, a thicker encapsulation facilitates overall low losses. However, in the case of PEEK, this effect reaches saturation after a thickness of 2 mm, where the losses are approximately similar for a thickness of 3 mm. It was also noticed that PEEK is the kind of material, which can be handled and manufactured far more easily than Zirconia, thus being more suitable for IBBDs. In summary, low-loss encapsulation helps mitigate the loss by concentrating the near field in a low-loss region.

### 4.5. Effect on Antenna Bandwidth

In-body antennas are compact in size and subject to narrower bandwidth [78]. However, all the radiated power from a transmitter does not reach the receiver because of significant absorption and reflection by the biological tissue medium. In an in-body antenna, the absorbed power is commonly greater than the reflected power, which generally causes the bandwidth to be wider. This also causes lower radiation efficiency of the antenna. It is possible to reduce these losses by using bio-encapsulation—as discussed in the previous section—and impedance matching, making the bandwidth narrower. However, an in-body antenna with narrow bandwidth suffers from frequency detuning inside the biological tissue environment. Therefore, a cautious consideration is necessary to solve this issue. The bandwidth of the in-body antenna can be improved by using a thicker substrate. In Ref. [85], the bandwidth of an implantable monopole antenna is improved by connecting a strip line with U-shaped ground. In Ref. [86], the ground plane of the implantable PIFA antenna is partly connected to a RFID circuit to enhance the bandwidth.

### 4.6. Effect on Antenna Radiation Pattern

The lossy medium present in a human body environment can cause broadening of the radiation pattern because of the reflection, refraction and scattering existing in or generated from the body tissues [78]. The radiation pattern of an in-body antenna would also be variable in the same medium if the mounting circumstances and in-body positions were different.

### 4.7. SAR Requirement

As explained in Section 2.6, SAR is used as a metric to guarantee the safety of biological tissues in the event of severe electromagnetic exposure. The standard SAR levels are maintained by IBBDs by considering low output power. In general, the specific absorption (*SA*) per pulse can be calculated by [87]

(13) $$SA=SAR\times T_{p}$$

where *T_p_* represents the pulse duration. The EM power absorbed by the biological tissue medium can raise the temperature of the tissue. It must be noted that the temperature of the biological tissues adjacent to the implanted device should not rise more than 1–2 °C.

### 4.8. Effective Isotropic Radiated Power (EIRP)

A remarkable level of EIRP of the in-body antenna can be harmful to the biological tissues, and it can create interference with the nearby radio devices. The standardized limit of EIRP for an in-body antenna functioning in the MedRadio band is −16 dBm [88] and −36 dBm at 915 MHz for the ISM band [89]. In case the in-body antenna is used for data telemetry, the input power must be limited to alleviate damage to the tissues. If the in-body antenna is operating as a receiver, the external source of power must follow the aforementioned standards.

### 4.9. Powering

Continuous power delivery to the IBBDs is one of the foremost challenges for in-body antennas. The current battery technologies are an inefficient solution for such application due to their short lifetime [11,14]. Furthermore, batteries contain hazardous ingredients and necessitate a surgical operation to be replaced. Additionally, the power system of IBBDs must be lightweight and easy to fabricate to facilitate an easy movement of patients. It is also necessary to maintain the energy level of the system in the design of a powering system for IBBDs.

## 5. In-Body Antenna Applications

This section explains the range of applications of IBBDs (implantable, ingestible and injectable devices) implemented with different types of in-body antennas.

### 5.1. Pacemaker

**Figure 9:**
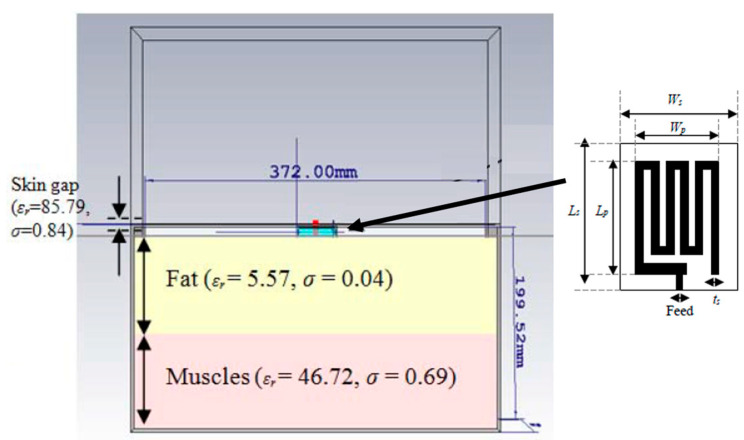
Compact meander line planar implantable antenna for a pacemaker [90]. Reproduced with permission from an open-access article from the *Indonesian Journal of Electrical Engineering and Computer Science* (CC-BY).

A compact meander line planar implantable antenna for a pacemaker application operating at 402.5 MHz with a bandwidth of 33.5% is presented by Samsuri et al. in Ref. [90]. The proposed antenna is implemented on a FR-4 substrate with *ε_r_* = 4.7 and tan *δ* = 0.025 with a size of 30.5 mm × 21.02 mm × 6.4 mm, as shown in Figure 9. The antenna performance is evaluated through simulation in a multi-layer human body model.

**Figure 10:**
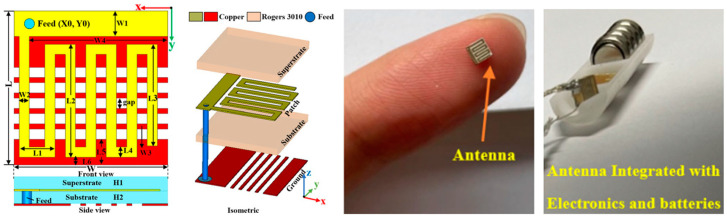
Tiny and compact implantable planar antenna for a wireless cardiac pacemaker [25]. Reproduced with permission from an open-access article from *Scientific Reports* (CC-BY).

Figure 10 shows a tiny and compact implantable planar antenna with the size of 3 mm × 3 mm × 0.5 mm as presented in Ref. [25] for a wireless cardiac pacemaker. Rogers 3010 is used as a superstrate and substrate where *ε_r_* = 10.2 and tan *δ* = 0.0023. The antenna is optimized and loaded with a defective slotted structure to improve the efficiency and overall performance of the antenna in an ISM frequency band of 2.4 to 2.48 GHz. The definite bandwidth of the antenna is 22%, with the peak gain of −24.9 dBi.

**Figure 11:**
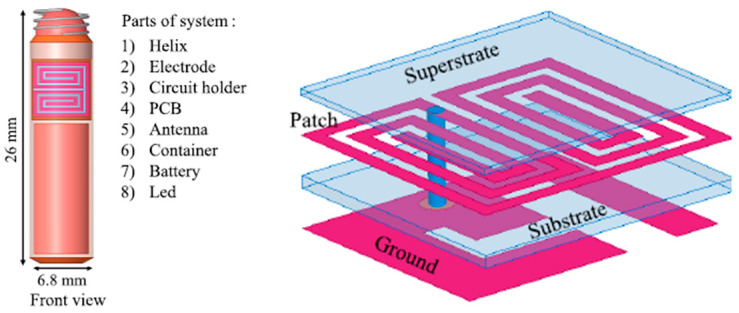

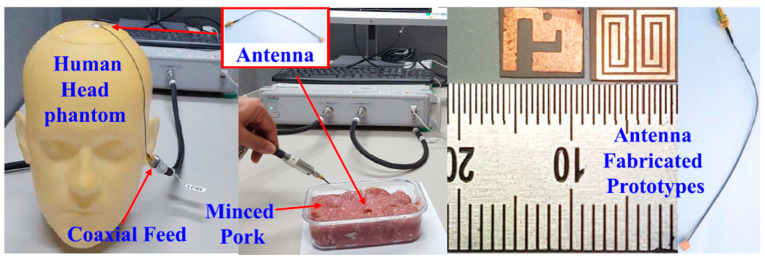
Triband spiral shaped antenna and its test setup in minced pork [68]. Copyright © *IEEE*.

A triband spiral shaped implantable antenna is presented by Shah et al. in Ref. [68] with slotted ground operating at 402 MHz, 1.6 GHz and 2.45 GHz for a leadless pacemaker system. The size of the antenna is 7 mm × 6.5 mm × 0.377 mm, where Rogers RT/Duroid 6010 with *ε_r_* = 10.2 and tan *δ* = 0.0035 is utilized as a superstrate and substrate, as shown in Figure 11. The gains of the antenna at the three different frequencies are −30.5 dBi, −22.6 dBi, −18.2 dBi, respectively, with bandwidths of 36.8%, 10.8% and 3.4%, respectively.

### 5.2. Blood Pressure Monitoring Implant

The frequent rise and drop in blood pressure can originate a stroke or severe cardiovascular disease for patients, which necessitates the requirement for an accurate blood pressure monitoring system in a healthcare space.

**Figure 12:**
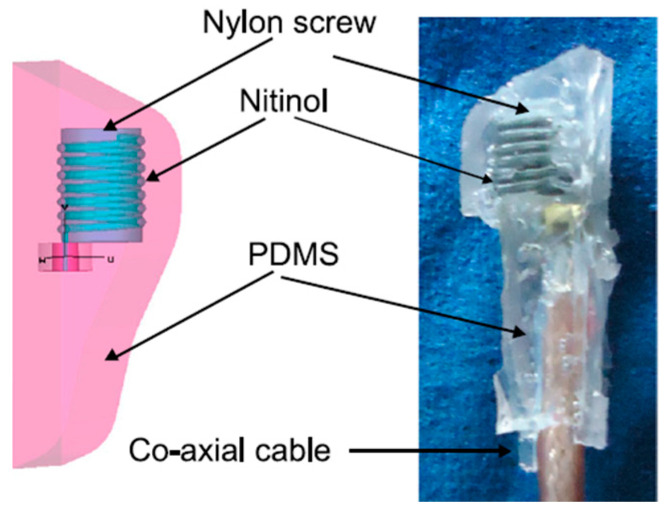
Helical wire antenna for blood pressure monitoring implant [23]. Reproduced with permission from an open-access article from *Biomedical Microdevices* (CC-BY).

The measurement of blood pressure through an implantable antenna system embedded into the heart would be an outstanding solution for risky heart patients. In Ref. [23], a pseudo-normal-mode helical antenna is presented with a poly-siloxane (PDMS) insulation layer, as shown in Figure 12. The operation frequency of the antenna is 863–870 MHz with a size of 3 mm in diameter and 9.44 mm in height. The antenna is built with a 0.33 mm diameter nitinol wire. The implant antenna and sensor are put inside the left ventricle and subjected to experimentation with a pig, as shown in Figure 8. This antenna can provide a maximum radiation efficiency of −27 dB and directivity of 2.65 dBi.

**Figure 13:**
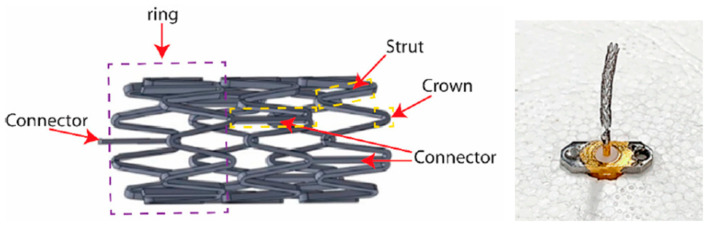
Smart stent antenna for intravascular monitoring [91]. Reproduced with permission from an open-access article from MDPI *Sensors* (CC-BY).

A smart stent antenna is presented in Ref. [91] for intravascular monitoring, as shown in Figure 13. The commonly used L-605 Cobalt–Chromium (Co–Cr) alloy is used as the material for the stent. The diameter and length of the stent are 2 mm and 18 mm, respectively. This antenna can achieve a gain of 1.38 dBi and a radiation efficiency of 74.5% at a resonant frequency of 2.07 GHz.

### 5.3. Brain Implant

**Figure 14:**
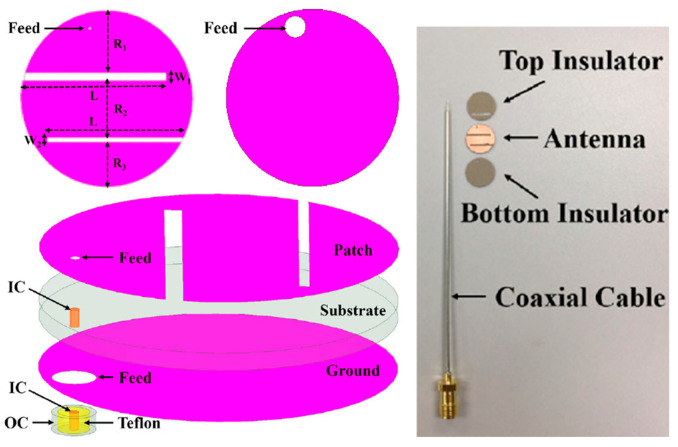
Miniaturized planar implantable antenna for a brain implant [92]. Reproduced with permission from an open-access article from *IEEE Access* (CC-BY).

Figure 14 shows a miniaturized planar implantable antenna presented in Ref. [92] with an operation frequency of 2.4 GHz. The approximate size of the antenna is 10 mm × 10 mm × 1.5 mm, and it is manufactured with Taconic RF-35 with *ε_r_* = 3.5 and tan *δ* = 0.0018. The achieved bandwidth of the antenna is 14.9%, with the peak gain of −20.75 dBi.

In Ref. [93], a 2.4 or 4.8 GHz planar implantable antenna with modified E-shape is presented, built with a Rogers TMM13i substrate with *ε_r_* = 12.2 and tan *δ* = 0.0019. The size of the implantable antenna is 10 mm × 8.7 mm × 0.76 mm. The maximum SAR achieved is 69 mW/Kg for 10 g of tissue.

The antenna presented in Ref. [68] is also compatible with a pacemaker application for a brain implant.

A novel flexible moon-shaped slot implantable antenna operating at 2.45 GHz frequency is presented for neural recording systems and brain implants in Ref. [94]. The size of the antenna is 8 mm × 9 mm × 0.2 mm, fabricated with a RO4003C substrate with *ε_r_* = 3.48 and tan *δ* = 0.0027. The peak gain achieved is approximately −13 dBi at 2.45 GHz. The maximum SAR achieved is less than 1 W/kg for 1 g of tissue.

### 5.4. Intracranial Pressure

**Figure 15:**
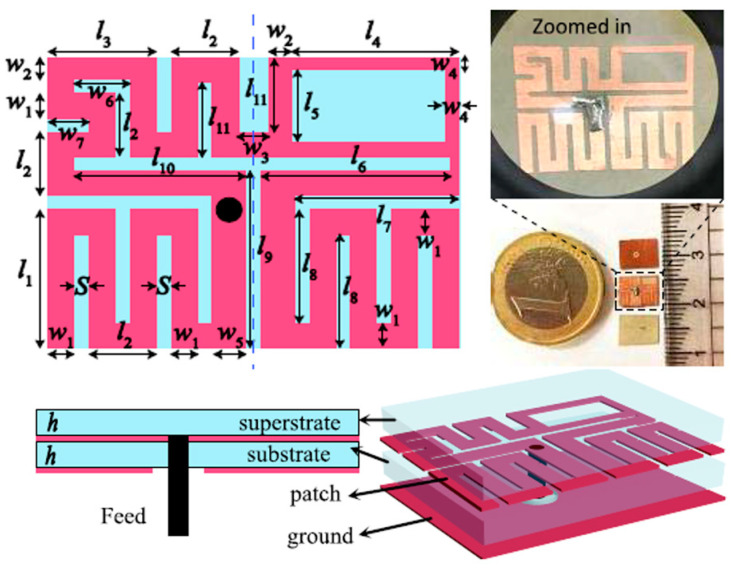
Miniaturized planar implantable antenna for intracranial pressure [95]. Copyright © *IEEE*.

Figure 15 shows a miniaturized planar implantable antenna proposed by Shah et al. for intracranial pressure monitoring at 915 MHz and 2.45 GHz [95]. The proposed antenna has a size of 8 mm × 6 mm × 0.5 mm and utilizes Rogers 6010 as the substrate with *ε_r_* = 10.2 and tan *δ* = 0.0023. Biocompatibility is confirmed by the ceramic alumina encapsulation. The antenna achieved a gain and bandwidth of −28.5 dBi and 9.84% at 915 MHz, respectively, and −22.8 dBi and 8.57% at 2.45 GHz, respectively. To achieve the safety limit of 2 W/kg for SAR_10g_, the maximum allowable input power is 17.12 mW at 915 MHz and 20.6 mW at 2.45 GHz.

**Figure 16:**
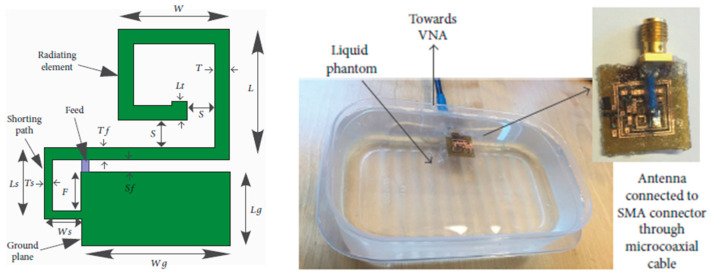
Coplanar miniature antenna for intracranial pressure tested in liquid phantom [96]. Reproduced with permission from an open-access article from the *International Journal of Antennas and Propagation* (CC-BY).

A coplanar miniature antenna with the size of 6 mm × 5 mm × 1 mm is presented in Ref. [96] with an operation frequency of 2.45 GHz, as shown in Figure 16. Khan et al. claimed to use low-permittivity polyimide as a flexible substrate for the proposed antenna. This antenna can provide a peak gain of −19.63 dBi and maximum SAR of 10 mW/kg in brain tissue.

In Ref. [97], a PIFA antenna design technique was explained for head-implanted medical devices, including intracranial pressure application. In this work, Rogers RO 3210 is selected as the substrate with *ε_r_* = 10.2 and tan *δ* = 0.003. The antenna is 12 mm in diameter and 1.8 mm in width. It was designed to operate at 402, 433, 868 and 915 MHz. It can achieve a SAR of 2 W/kg for 10 g of tissue when the input power is 4.927 mW. It can achieve a peak gain of −36.90, −35.99, −35.14 and −32.94 dB at 402, 433, 868 and 915 MHz, respectively.

A wireless power receiver spiral antenna with dimensions of 12.88 mm × 13.46 mm × 0.05 mm is presented by Waqas et al. in Ref. [98] for an intracranial pressure implant. The antenna is made on a flexible polyimide substrate with *ε_r_* = 3.3 and tan *δ* = 0.002. The operating frequency of the antenna was selected as 11 MHz, where a −2.17 dB measurement peak gain was achieved. The wireless power transfer efficiency was 1.18%.

### 5.5. Glucose Monitoring and Sensing

**Figure 17:**
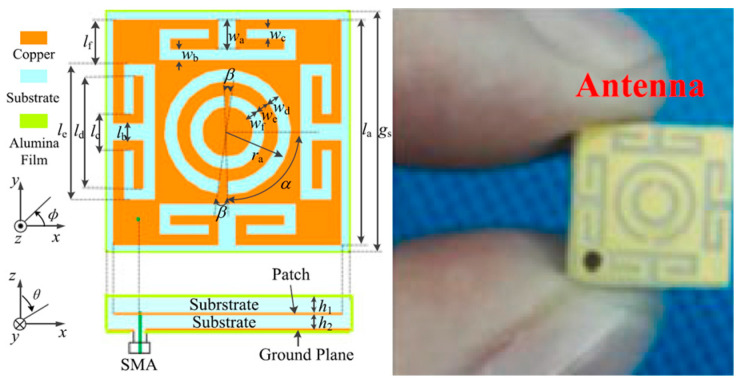
Proposed wide-beamwidth circularly polarized implantable antenna for glucose monitoring presented in Ref. [99]. Copyright © *IEEE*.

In Ref. [99], a miniaturized single-fed wide-beamwidth circularly polarized implantable antenna working in the ISM band (2.40–2.48 GHz) is presented for subcutaneous real-time glucose level monitoring application. Figure 17 shows the proposed antenna with the dimensions of 8.5 mm *×* 8.5 mm *×* 1.27 mm employing four C-shaped slots and a complementary split-ring resonator (CSRR). Meanwhile, by adjusting the slits of the CSRR, circular polarization is realized. In this work, Rogers 3210 is selected as the substrate with *ε_r_* = 10.2 and tan *δ* = 0.003. The simulation results with a three-layer phantom demonstrate that the impedance bandwidth is 12.2%, with a peak gain of −17 dBi.

**Figure 18:**
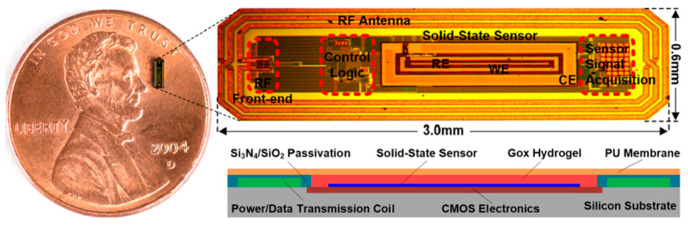
Injectable glucose sensing device with spiral antenna [100]. Reproduced with permission from an open-access article from *Scientific Reports* (CC-BY).

Mujeeb-U-Rahman et al. in Ref. [100] presented a spiral antenna for wireless power and data telemetry for an injectable glucose sensing device. The size of the antenna is 3 mm *×* 0.6 mm. It is operating at the 900 MHz ISM band, with the peak power gain of less than −30 dB. The power transfer efficiency of the inductive link is 0.1%. The antenna is built on a silicon substrate through a photolithography process, as shown in Figure 18.

### 5.6. Orthopedic Implant Infection Monitoring

**Figure 19:**
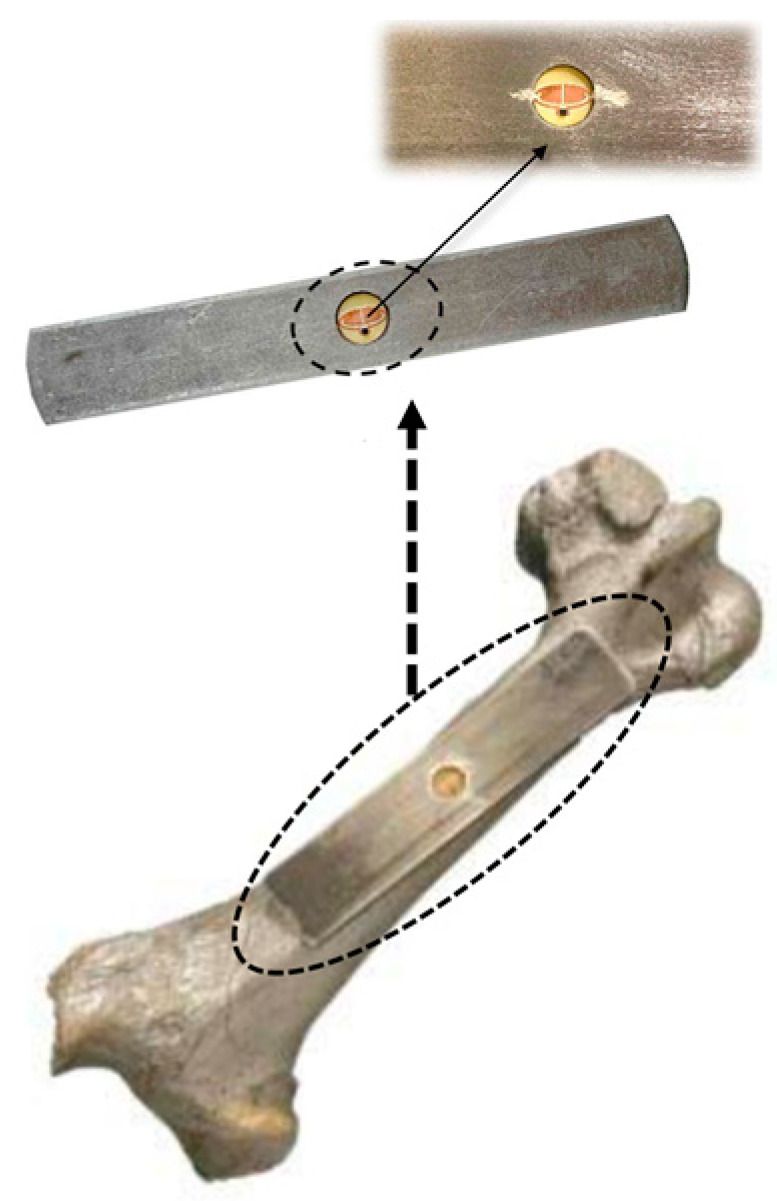
Planar implantable antenna for an UHF RFID-based orthopedic implant [101]. Copyright © *IEEE*.

A planar implantable antenna for monitoring infection in an orthopedic implant is presented in Ref. [101] for the 860 to 960 MHz RFID ultra-high frequency (UHF) band, as shown in Figure 19. The size of the antenna is 14 mm *×* 6 mm *×* 3 mm. It is fabricated on a FR4 substrate and achieves a peak gain of −22 dBi.

### 5.7. Cochlear Implant

**Figure 20:**
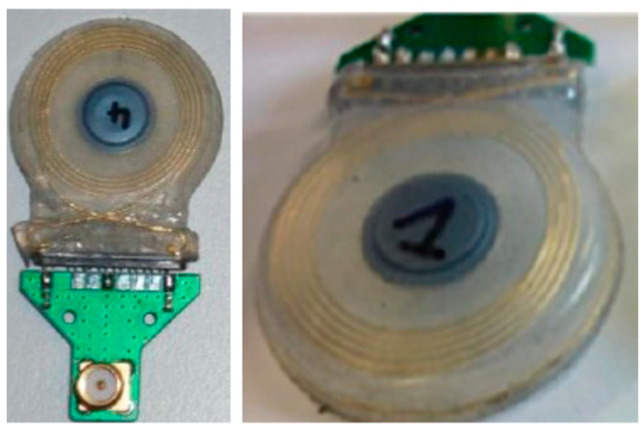
Folded loop wire antenna for a cochlear implant [102]. Reproduced with permission from Attribution-NoDerivaties 4.0 International shared at CSEM archive.

A folded loop antenna built with a metal wire with the size of 38 mm *×* 38 mm *×* 2.2 mm and a wire radius of 0.3 mm is presented in Ref. [102] for a cochlear implant, as shown in Figure 20. The operating frequency of the proposed antenna is 2.45 GHz, with a bandwidth of 8.57%. It realizes a gain of −0.1 dBi.

### 5.8. Retinal Implant

A PIFA antenna with a MedRadio (401–406 MHz) band is presented by Orfeas and Nikita in Ref. [103] for a retinal implant. The antenna diameter is approximately 12 mm with a thickness of 1.8 mm in PEEK encapsulation. In this work, Rogers 3210 is selected as the substrate with *ε_r_* = 10.2 and tan *δ* = 0.003. This antenna achieves a peak gain of −36.82 dBi with a bandwidth of 3.4% in PEEK encapsulation. It can achieve a SAR of 2 W/kg for 10 g of tissue when the input power is 21 mW in PEEK encapsulation.

### 5.9. Capsule Endoscopy

Capsule endoscopy (CE) is the most common ingestible device, which is used for diagnosis and monitoring of different gastrointestinal (GI) disorders. A wide range of in-body antennas used for CE application over the last few years are described below.

**Figure 21:**
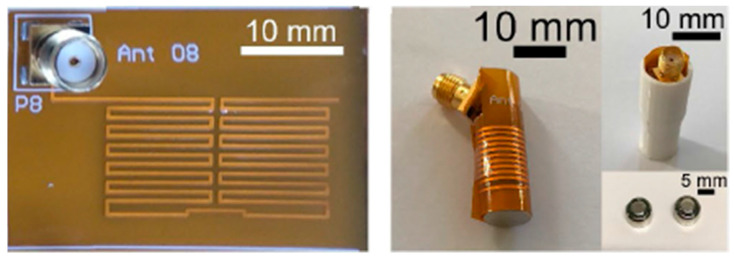
Fabricated planar meandering antenna with SMA connection [104]. Reproduced with permission from an open-access article from *IEEE Access* (CC-BY).

A planar meandering antenna fabricated on a 0.1 mm thick polyimide flexible substrate (with *ε_r_* = 3.5 and tan *δ* = 0.0027) with 0.035 mm copper thickness is presented in Ref. [104], as shown in Figure 21. The proposed antenna has dimensions of 28 mm *×* 12 mm, working with an operation frequency of 433 MHz. The measured peak gain is −39 dBi.

In Ref. [105], a circularly polarized (CP) helical implantable antenna is proposed. The proposed antenna operates at 2.4 GHz frequency with a gain of −19.83 dBi and a bandwidth of 290 MHz. A perfect electric conductor (PEC) is used to build and simulate this antenna with a 6.6 mm diameter and 8.85 mm length, with an approximate wire thickness of 0.4 mm. Furthermore, the antenna covers the cylindrical shape of the capsule with a diameter of 7.06 mm and length of 25 mm.

**Figure 22:**
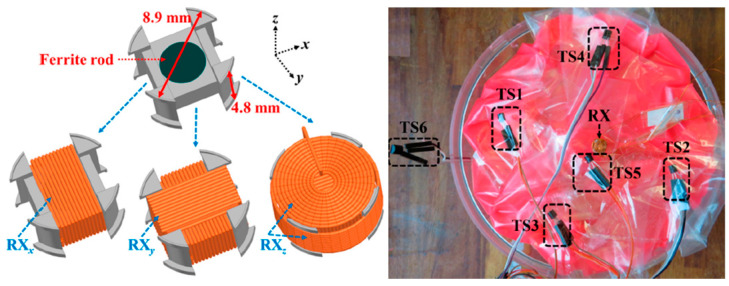
Three-dimensional wireless power transfer receiver coil for capsule endoscopy tested in gel phantom [61]. Reproduced with permission from an open-access article from MDPI *Micromachines* (CC-BY).

A 3D wireless power transfer receiver coil of 8.9 mm in diameter and 4.8 mm in thickness is presented in Ref. [61], as shown in Figure 22. The proposed 3D coil is built with a 0.2 mm copper wire. It achieves 0.7% power transfer efficiency (PTE) and a SAR of 66 mW/kg for 10 g of tissue at 1 MHz operation frequency.

A conformal antenna with a frequency of 402, 433, 915 and 2450 MHz is presented with an achieved gain of −32.5, −30.4, −17.9 and −19.0 dBi, respectively [106]. The proposed antenna is printed on a 0.17 mm thick flexible Kapton substrate with *ε_r_* = 3.5 and tan *δ* = 0.0027. It has a size of 12 mm *×* 6 mm. The bandwidth of the antenna is reported to be 2.95 and 3.33 GHz.

**Figure 23:**
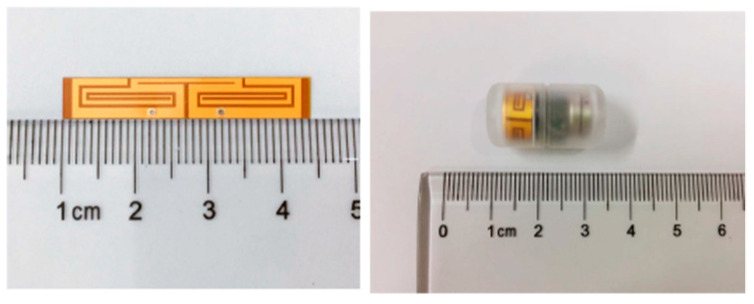
Conformal differentially fed antenna built in a capsule [107]. Copyright © *IEEE*.

A similar conformal differentially fed antenna with an operation frequency of 915 MHz is presented in Ref. [107]. The proposed antenna has dimensions of 32 mm *×* 5.8 mm, as shown in Figure 23, and it is printed on a flexible polyimide substrate (with *ε_r_* = 3.5 and tan *δ* = 0.008) of 0.15 mm in thickness. The antenna achieves a peak gain of −21 dBi and a bandwidth of 8.9% tested in the phantom under 50 mm depth.

**Figure 24:**
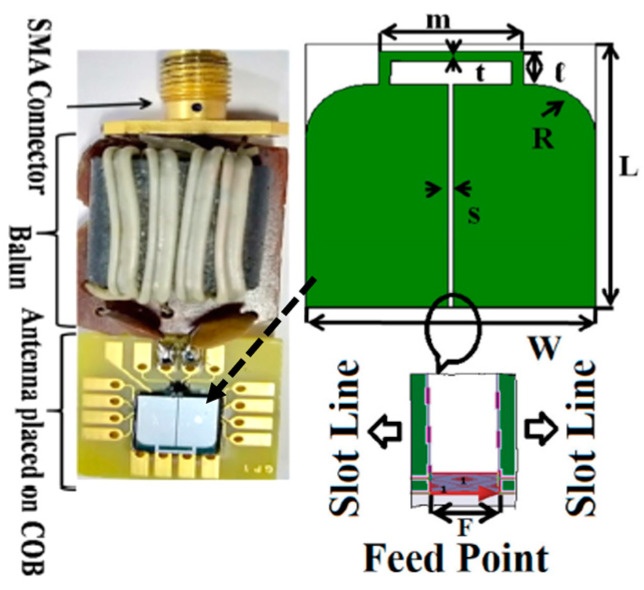
Miniaturized slot line fed antenna for capsule endoscopy [108]. Reproduced with permission from an open-access article from *IET Microwaves, Antennas & Propagation* (CC-BY).

Figure 24 shows a slot line fed antenna with the size of 7 mm *×* 7 mm constructed on a silicon substrate of approximately 0.675 mm in thickness with *ε_r_* = 11.9, which is presented in Ref. [108]. The proposed antenna is operating at a 915 MHz frequency. It can achieve a peak gain of −35.5 dBi and a bandwidth of 300 MHz, as tested in colon phantom. Furthermore, it achieves a SAR of 8 mW/kg for 1 g of colon tissue.

**Figure 25:**
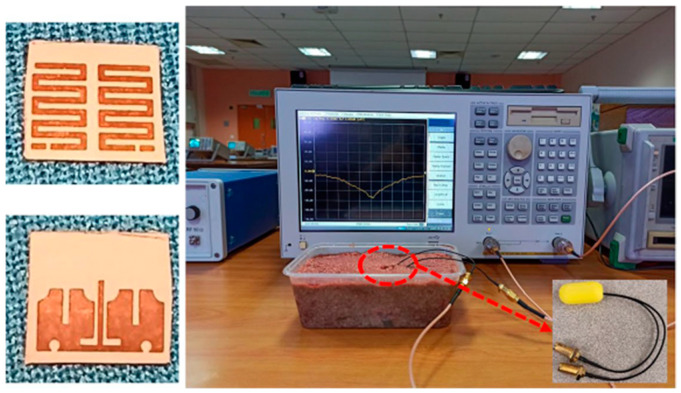
Fabricated MIMO antenna tested inside minced meat [109]. Reproduced with permission from an open-access article from *Scientific Reports* (CC-BY).

A wideband multiple-input–multiple-output (MIMO) compact antenna for ingestible capsules is presented in Ref. [109], as shown in Figure 25. The size of the antenna is 5 mm *×* 4.2 mm *×* 0.12 mm, operating at a 2.45 GHz frequency. The proposed MIMO antenna is manufactured on a Rogers RO3010 substrate with *ε_r_* = 10.2 and tan *δ* = 0.0022. It maintains a peak gain of −20.6 dBi and a bandwidth of 25%. It can achieve a SAR of 2 W/kg for 10 g of tissue when the input power is 3.97 mW.

### 5.10. Cell Rover

**Figure 26:**
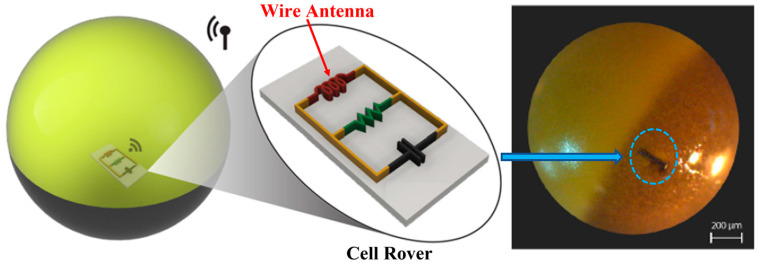
Cell rover with wire antenna inserted into the cell membrane [110]. Reproduced with permission from an open-access article from *Nature Communications* (CC-BY).

In Ref. [110], a sub-micrometer-sized injectable wire antenna is presented for the purpose of smart sensing, modulation, as well as energy harvesting to power in-cell nano-electronic computing. Figure 26 shows the proposed cell rover device, which can help understand the cell biology for different diagnostic and therapeutic applications. The receiver coil antenna is built with an American Wire Gauge (AWG) 47 wire, with a thickness of 0.0355 mm. The diameter and length of the coil are 2 mm and 1 mm, respectively. The proposed antenna operates at a 4.5 MHz frequency. It can achieve a PTE of 0.63% and a SAR of 0.0226 mW/kg for 10 g of tissue.

### 5.11. Pharmacology and Optogenetics

**Figure 27:**
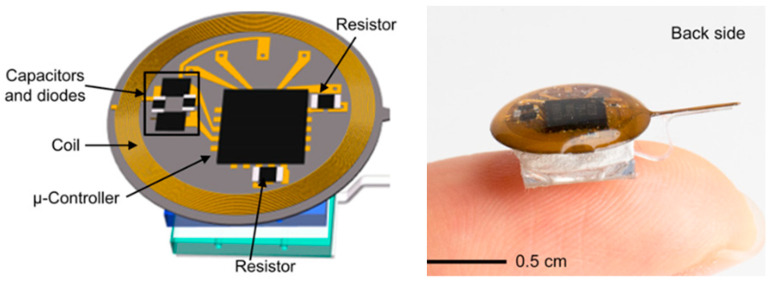
Wireless, battery-free, injectable microsystems for programmable pharmacology and optogenetics applications [111]. Reproduced with permission from an open-access article from *PNAS-Biological Sciences* (CC-BY).

In Ref. [111], an injectable IBBD is presented for pharmacology and optogenetics applications, as shown in Figure 27. Pharmacology and optogenetics are widely used in neuroscience research to study the central and peripheral nervous systems. This IBBD includes a spiral antenna of approximately 5 mm in diameter, operating at a 13.56 MHz frequency. The spiral antenna is fabricated on a flexible sheet of copper clad polyimide.

Table 1, Table 2 and Table 3 summarize the different types of in-body antennas for implantable, ingestible and injectable IBBD applications, respectively, discussed in this section, highlighting their structure (type and substrate) and key performance parameters (size, gain, −10 dB bandwidth and SAR).

## 6. Some Future Research Challenges

This section describes some of the limitations of the current in-body antenna designs and future challenges, which need to be resolved to improve performance.

1. Generally, the coupling of in-body antennas with lossy tissue causes the absorption of the EM wave in the near reactive and far field, which is commonly not considered in the design phase. This results in a significant reduction in the radiation efficiency and peak gain, causing inefficient antenna operation. This is inevitable in the far field. However, it is possible to reduce the absorption of the EM wave by covering the in-body antenna with biocompatible material in the near field. Therefore, designing in-body antennas with biocompatibility covering the near field will be a conceivable future research challenge.
2. The human body is formed with inhomogeneous biological tissues and organs. Furthermore, the characteristics and dimensions of biological tissues vary every so often, including by gender. Therefore, the detuning effect of the in-body antenna inside the human body is considered as one of the primary research and design IBBD applications. To date, in-body antenna design and experiments are mostly restricted only to a single tissue environment, which will be a noteworthy shortcoming for diverse biological tissue environments. Therefore, the upcoming in-body antenna research focus must be the investigation of diverse biological environments for efficient in-body antenna operation.
3. The implantation of a device operating at radio frequency inside the biological tissue may lead to a severe long-term health problem due to radiative power absorption. Therefore, an effective and optimized in-body antenna design with a SAR value limit as standard and an appropriate selection of biocompatible materials will be the key future research investigation.
4. Traditional antenna miniaturization techniques tend toward narrow operational bandwidths. Such narrowband operation can cause a detuning effect of the in-body antenna inside the biological environment. Biocompatible encapsulation of the in-body antenna can be utilized to increase the radiation efficiency and gain. However, this inflates the overall IBBD thickness. Therefore, an antenna design technique with acceptable operational bandwidth, radiation efficiency and gain is still a challenging matter in IBBDs. Increasing the operation frequency band can increase the miniaturization scale. However, this increases the loss and tissue absorption, which introduces additional design challenge.
5. Lastly, battery-powered IBBDs have a limited lifetime and bulky dimension, which can cause insufficiency in an in-body antenna power system. Furthermore, the replacement of the battery through a surgical procedure is complex and costly. Therefore, designing power-efficient IBBDs for in-body antennas is a crucial design challenge for the future.

## 7. Conclusions

A comprehensive review is presented for in-body antennas for IBBDs (implantables, ingestibles, injectables). Designing an in-body antenna operating in a harsh bio-tissue environment is a challenging task, where several specifications are required to be considered, including the operation frequency, band selection, size, performance (gain, efficiency, radiation pattern), biocompatibility and patient safety. Widely used miniaturization techniques, such as high-permittivity substrates, lengthier current flow path on the radiator, inductive or capacitive loading, pin shorting, higher operating frequencies, ground plane modifications and use of metamaterials, are discussed, and each technique is evaluated in terms of its merits and issues. This study shows that despite having the superior potential of miniaturization, higher frequency usage suffers from significant losses, which requires additional investigations in this domain, as radio wave exposure studies and research works at these frequencies are not yet well established. Moreover, this paper reviews the antenna design and manufacturing process and illustrates the antenna testing procedures, including in vitro and in vivo testing. This paper also summarizes several existing in-body antenna designs, including planar, PIFA, wire, conformal, spiral, slotted and MIMO structures, and classifies antennas according to the IBBD applications. Although the selection of an antenna type depends on specific applications, patch-type planar structures have been more commonly adapted by researchers, as shown in Table 1. The study of patient safety is one of the primary requirements for IBBDs, which is strictly administered through the SAR and EIRP limits. Table 1 shows that most researchers ignore this step while designing in-body antennas for IBBDs, which is alarming, as it fails to demonstrate the suitability of the proposed antennas for in-body applications. In-body antennas are obligated to meet these regulations while considering the bio-tissue environment as the key testing platform and further confirming the safety profile through utilization of biocompatible materials. Finally, the article concludes with listing some of the drawbacks and limitations of existing in-body antenna technology and the forthcoming research challenges in this area.

## Figures and Tables

**Table 1:** Summary of different types of in-body antennas used in implantable IBBD applications.

Antenna	Ref, Application	Frequency	Size (mm × mm × mm)	Substrate/Material	Gain (dBi)	Bandwidth	SAR_10g_ mW/kg
Planar	[90], Pacemaker	402.5 MHz	30.5 × 21.02 × 6.4	FR-4, *ε_r_* = 4.7, tan *δ* = 0.025	-	33.5%	-
	[25], Pacemaker	2.4 to 2.48 GHz	3 × 3 × 0.5	Rogers 3010, *ε_r_* = 10.2, tan *δ* = 0.0023	−24.9	22%	147.7
	[92], Brain implant	2.4 GHz	10 × 10 × 1.5	Taconic RF-35, *ε_r_* = 3.5, tan *δ* = 0.0018	−20.75	14.9%	0.3
	[93], Brain implant	2.4/4.8 GHz	10 × 8.7 × 0.76	Rogers TMM13i, *ε_r_* = 12.2, tan *δ* = 0.0019	-		69
	[95], Intracranial pressure	915 MHz, 2.45 GHz	8 × 6 × 0.5	Rogers 6010, *ε_r_* = 10.2, tan *δ* = 0.0023	−28.5, −22.8	9.84%, 8.57%	2000
	[96], Intracranial pressure	2.45 GHz	6 × 5 × 1	Polymide	−19.63	-	10
	[101], Orthopedic	860 to 960 MHz	14 × 6 × 3	FR4	−22	-	-
PIFA	[97], Intracranial pressure	402, 433, 868 and 915 MHz	Dia = 12 mm, Thick = 1.8 mm	Rogers RO 3210, *ε_r_* = 10.2, tan *δ* = 0.003	−36.90, −35.99, −35.14 and −32.94 dB	-	2000
	[103], Retinal implant	401–406 MHz	Dia = 12 mm, Thick = 1.8 mm	Rogers RO 3210, *ε_r_* = 10.2, tan *δ* = 0.003	−36.82	3.4%	2000
Wire	[23], Blood pressure monitoring	863 to 870 MHz	Dia = 3 mm Length = 9.44 mm	Nilton wire, 0.33 mm thick	Directivity = 2.65 dBi	-	-
	[91], Intravascular monitoring	2.07 GHz	Dia = 2 mm, Length = 18 mm	Co–Cr alloy	−1.38	-	-
	[102], Cochlear implant	2.45 GHz	38 × 38 × 2.2	Metal wire, 0.3 mm thick	– 0.1	8.57%	-
Spiral	[68], Brain implant and pacemaker	402 MHz, 1.6 GHz and 2.45 GHz	7 × 6.5 × 0.377	Rogers RT/Duroid 6010, *ε_r_* = 10.2, tan *δ* = 0.0035	−30.5, −22.6, −18.2	36.8%, 10.8% and 3.4%	-
	[98], Intracranial pressure	11 MHz	12.88 × 13.46 × 0.05	Flexible polyimide, *ε_r_* = 3.3, tan *δ* = 0.002	–2.17 dB	-	-
Slot	[94], Brain implant	2.45 GHz	8 × 9 × 0.2	RO4003C, *ε_r_* = 3.48, tan *δ* = 0.0027	−13	-	<1 W/kg for 1 g of tissue
	[99], Glucose monitoring	2.40 to 2.48 GHz	8.5 × 8.5 × 1.27	Rogers RO 3210, *ε_r_* = 10.2, tan *δ* = 0.003	−17	12.2%	-

**Table 2:** Summary of different types of in-body antennas used in ingestible IBBD applications.

Antenna	Ref, Application	Frequency	Size (mm × mm × mm)	Substrate/Material	Gain (dBi)	Bandwidth	SAR_10g_ mW/kg
Planar	[104], CE	433 MHz	28 × 12 × 0.035	Polyimide, *ε_r_* = 3.5, tan *δ* = 0.0027	−39 dBi	-	-
Wire	[105], CE	2.4 GHz	Dia = 6.6 mm, Length = 8.85 mm	PEC wire, 0.4 mm thick	−19.83	12%	
	[61], CE	1 MHz	Dia = 8.9 mm, Thick = 4.8 mm	Copper wire, 0.2 mm thick	0.7% PTE	-	66
Conformal	[106], CE	402 MHz, 433 MHz, 915 MHz and 2.45 GHz	12 × 6 × 0.17	Kapton substrate,*ε_r_* = 3.5, tan *δ* = 0.0027	−32.5, −30.4, −17.9 and −19.0	-	-
	[107], CE	915 MHz	32 × 5.8 × 0.15	Polyimide, *ε_r_* = 3.5, tan *δ* = 0.008	−21	8.9%	
Slot	[108], CE	915 MHz	7 × 7 × 0.675	Silicon substrate, *ε_r_* = 11.9	−35.5 dBi	32.8%	8 mW/kg for 1 g of tissue
MIMO	[109], CE	2.45 GHz	5 × 4.2 × 0.12	Rogers RO 3010, *ε_r_* = 10.2, tan *δ* = 0.0022	−20.6	25%	2000

**Table 3:** Summary of different types of in-body antennas used in injectable IBBD applications.

Antenna	Ref, Application	Frequency	Size (mm × mm × mm)	Substrate/Material	Gain (dBi)	Bandwidth	SAR_10g_ mW/kg
Spiral	[100], Glucose sensing	900 MHz	3 × 0.6	Silicon substrate (Photolithography process)	−30 dB	-	-
	[111], Pharmacology and optogenetics	13.56 MHz	Dia = 5 mm	Polyimide	-	-	-
Wire	[110], Cell rover	4.5 MHz	2 × 1	AWG 47 (0.0355 mm)	-	0.63%	0.0226

## Data Availability

No new data were created in this research.
